# Correction: Zhou et al. Flexible and Effective Preparation of Magnetic Nanoclusters via One-Step Flow Synthesis. *Nanomaterials* 2022, *12*, 350

**DOI:** 10.3390/nano15181389

**Published:** 2025-09-10

**Authors:** Lin Zhou, Lu Ye, Yangcheng Lu

**Affiliations:** State Key Laboratory of Chemical Engineering, Department of Chemical Engineering, Tsinghua University, Beijing 100084, China; zhoul20@mails.tsinghua.edu.cn (L.Z.); jennsie16@163.com (L.Y.)

Following publication, concerns were raised regarding the relevance of a few references in this publication [1]. Adhering to our standard procedure, the Editorial Board conducted an investigation which determined that references [3,4,6], as suggested by one of the peer reviewers, are irrelevant to the content of the article. As a result, following discussion with the authors, the Editorial Board has decided to remove the irrelevant citations [3,4,6] from this publication [1].

3. Tishkevich, D.I.; Vorobjova, A.I.; Vinnik, D.A. Formation and corrosion behavior of Nickel/Alumina nanocomposites. *Solid State Phenom.* **2020**, *299*, 100–106. https://doi.org/10.4028/www.scientific.net/SSP.299.100.
4. Tishkevich, D.I.; Vorobjova, A.I.; Trukhanov, A.V. Thermal Stability of Nano-Crystalline Nickel Electrodeposited into Porous Alumina. *Solid State Phenom.* **2020**, *299*, 281–286. https://doi.org/10.4028/www.scientific.net/SSP.299.281.
6. Dukenbayev, K.; Korolkov, I.V.; Tishkevich, D.I.; Kozlovskiy, A.L.; Trukhanov, S.V.; Gorin, Y.G.; Shumskaya, E.E.; Kaniukov, E.Y.; Vinnik, D.A.; Zdorovets, M.V.; et al. Fe_3_O_4_ Nanoparticles for Complex Targeted Delivery and Boron Neutron Capture Therapy. *Nanomaterials* **2019**, *9*, 494. https://doi.org/10.3390/nano9040494.

With this correction, the order of some references has been adjusted accordingly. The authors state that the scientific conclusions are unaffected. The publication and associated webpage have been updated accordingly. The Editorial Board is satisfied that the publication of this paper is in line with their expectations and MDPI’s Editorial Process policy.

## References

[1] Zhou L., Ye L., Lu Y. (2022). Flexible and Effective Preparation of Magnetic Nanoclusters via One-Step Flow Synthesis. Nanomaterials.

