# Supplementary material for: Flexible and Effective Preparation of Magnetic Nanoclusters via One-Step Flow Synthesis
Source: Nanomaterials (Basel). 2022 Jan 22;12(3):350. doi: 10.3390/nano12030350 (PMC8840485; doi:10.3390/nano12030350)
Supplement: Supplementary file 1 [file nanomaterials-12-00350-s001.zip › nanomaterials-1563486-supplementary.pdf]

nanomaterials

MDPI

Supplementary Materials

Flexible and Effective Preparation of Magnetic Nanoclusters via One-Step Flow Synthesis

Lin Zhou, Lu Ye and Yangcheng Lu *

[1] State Key Laboratory of Chemical Engineering, Department of Chemical Engineering, Tsinghua University, Beijing 100084, China; zhoul20@mails.tsinghua.edu.cn (L.Z); jennsie16@163.com (L.Y.)

* Correspondence: luyc@tsinghua.edu.cn; Tel.: +86-10-6277-3017

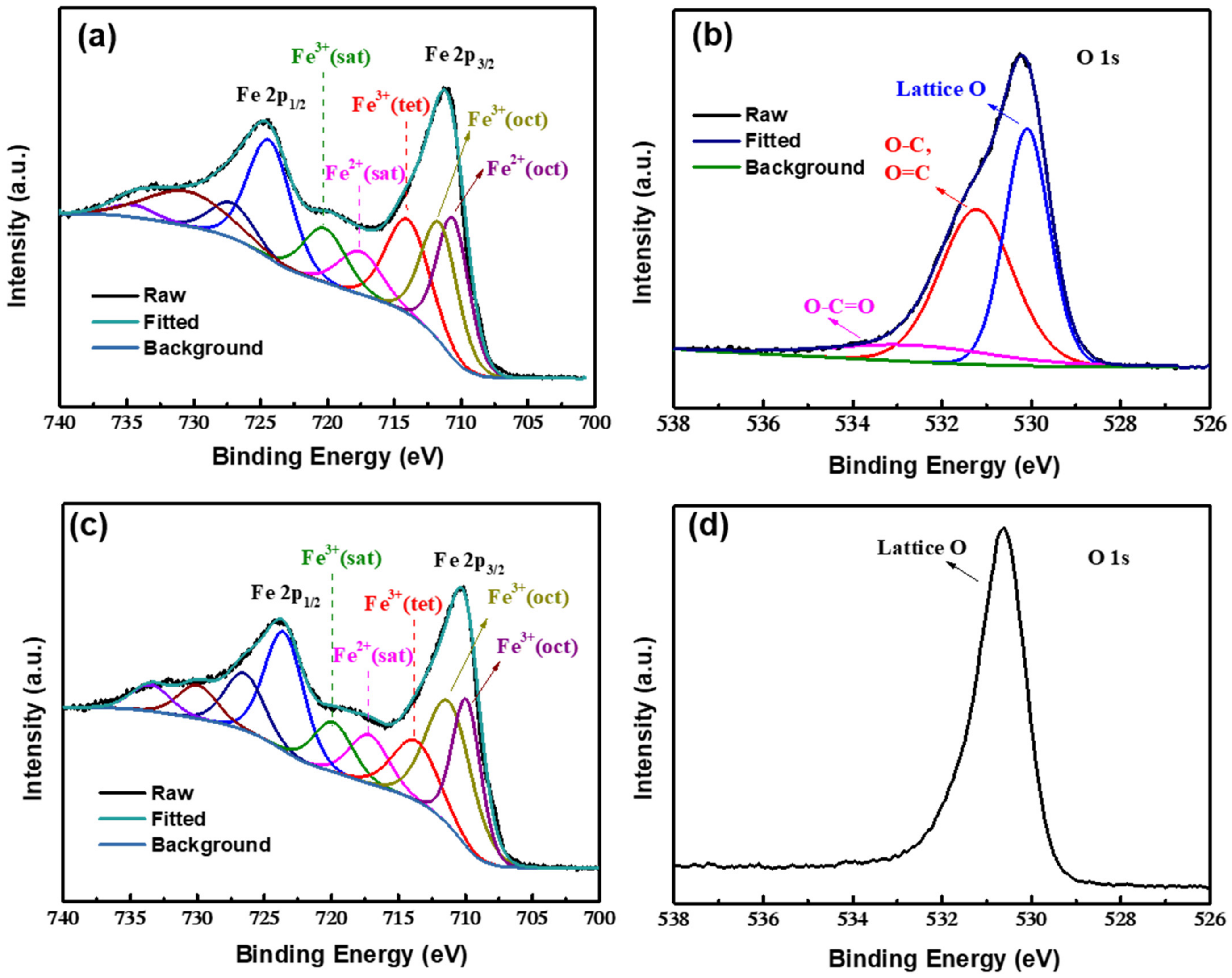

Figure S1. XPS spectra of bilayer OA-coated Fe_3O_4 nanoclusters for (**a**) Fe 2p, (**b**) O 1 s; and bare Fe_3O_4 nanoparticles under the same conditions for (**c**) Fe 2p, (**d**) O 1 s.

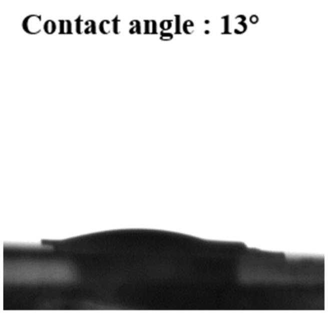

Figure S2. Contact angle of bilayer OA-coated Fe_3O_4 nanoclusters.

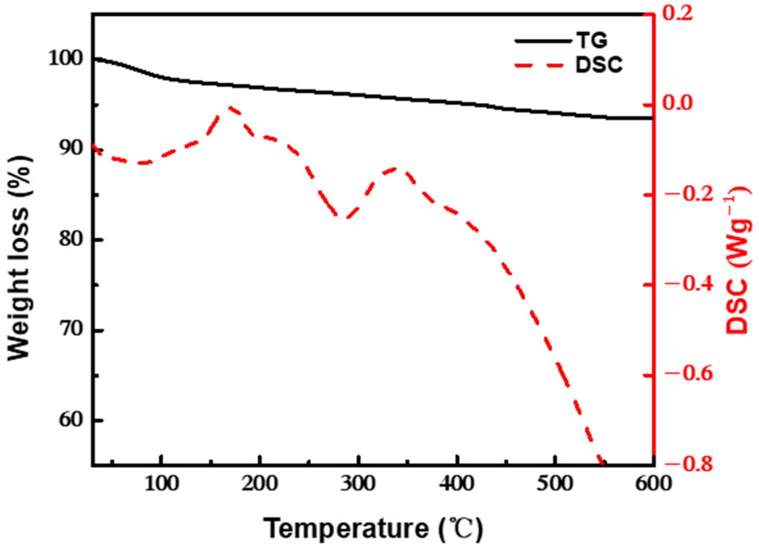

Figure S3. Thermogravimetric analysis of bare Fe_3O_4 nanoparticles under the same conditions.

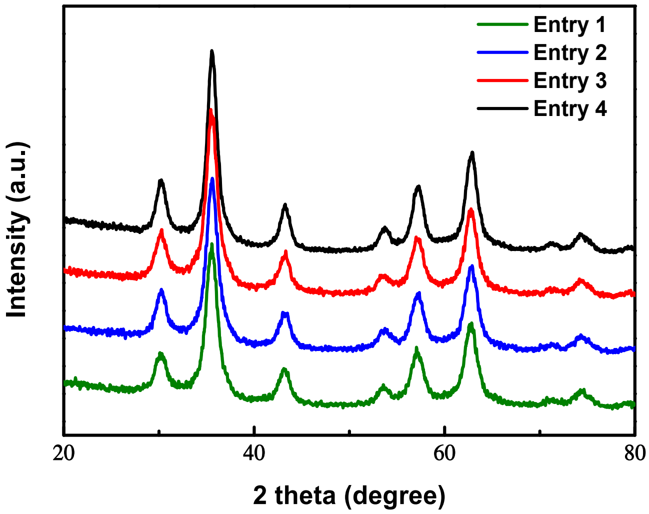

Figure S4. XRD patterns for nanoclusters obtained with different residence time 1.

Synthesis of bilayer OA-coated Fe_3O_4 nanoclusters in batch

The batch process was carried out in a 100 mL three-necked flask. Typically, 1.296 g $FeCl_3$ and 0.7972 g $FeCl_2{\cdot}4H_2O$ were dissolved in 20 mL of distilled water at room temperature, and then bubbled with pure nitrogen for 30 min to remove the dissolved oxygen. After the solution was heated to 60 °C with high-speed mechanical stirring (700 rpm), 20 mL of 7.3 M ammonia was rapidly poured into the solution for the formation of

nanoparticles. After 30 s of reaction, 20 mL ammonia solution containing 0.1 M OA was dropped into the solution, and a black–brown stable water-based magnetic fluid was formed after 60 mins of reaction.

From Figure S5, it could be found that the average hydraulic diameter of the clusters was 148 nm with a broader size distribution than those obtained via the continuous process. The TEM image also showed that the size and shape of the corresponding clusters varied considerably, and there were large aggregates in the field of view. In addition, under the same amount of OA, the final weight loss was 22.3 wt%, in which the amount of the inner chemical adsorbed layer of OA could reach 13.7 wt%, while the outer physical adsorbed layer of OA was only 8.6 wt% (Figure S6). This means that long-term stirring made OA achieve a relatively sufficient reaction, and basically complete the monolayer modification on the surface of the particles, triggering an unstable agglomeration process under this reaction condition. However, owing to the poor mixing performance of the batch process, the physical adsorption process between the two layers of OA was not quick enough to inhibit the growth of the modified clusters in time.

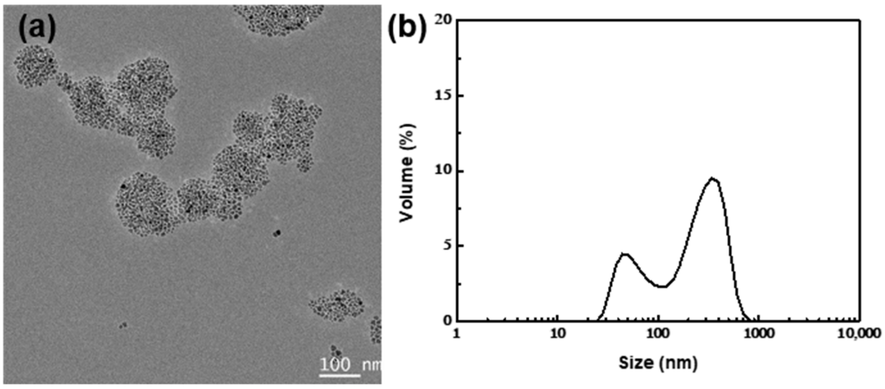

Figure S5. (**a**) TEM images and (**b**) size distribution of bilayer OA-coated Fe_3O_4 nanoclusters prepared in batch.

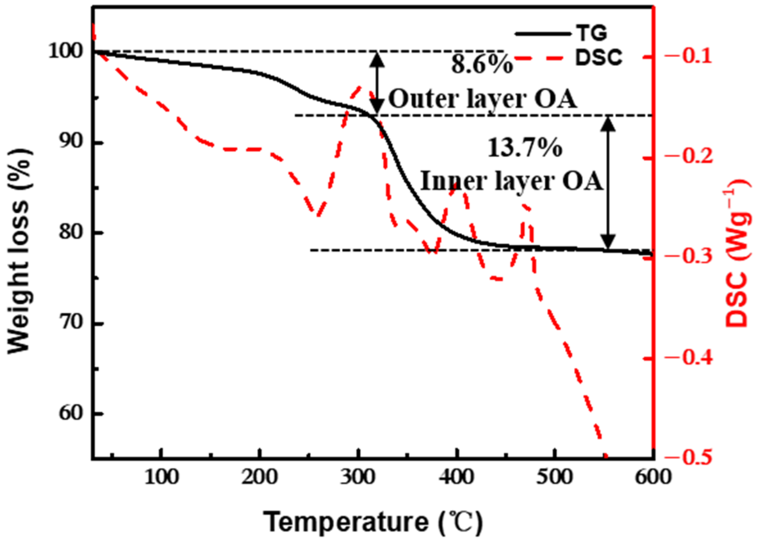

Figure S6. Thermogravimetric analysis of bilayer OA-coated Fe_3O_4 nanoclusters prepared in batch.
